# Acknowledgment to Reviewers of *Journal of Functional Biomaterials* in 2020

**DOI:** 10.3390/jfb12010006

**Published:** 2021-01-24

**Authors:** Journal of Functional Biomaterials Editorial Office

**Affiliations:** MDPI AG, St. Alban-Anlage 66, 4052 Basel, Switzerland

Peer review is the driving force of journal development, and reviewers are gatekeepers who ensure that *Journal of Functional Biomaterials* maintains its standards for the high quality of its published papers. Thanks to the cooperation of our reviewers, in 2020, the median time to first decision was 17 days and the median time to publication was 31 days. The editors would like to express their sincere gratitude to the following reviewers for their precious time and dedication, regardless of whether the papers were finally published:
Acquah, CalebLiu, Shyh-JiunAlam, Seemi TasnimLiu, Ting-YuAmaral, Isabel F.Liu, YizhiAmbu, RitaLizio, GiuseppeAmstutz, VéroniqueLondoño López, Martha ElenaAntunes, Joana C.Lops, DiegoArahira, TakaakiLukaszewska-Kuska, MagdalenaAsano, MasatakeLuo, ZishengBakowsky, UdoMaisch, TimBalhaddad, Abdulrahman A.Makvandi, PooyanBarbalinardo, MariannaMalemud, Charles J.Barbera, OrazioMarto, Carlos MiguelBashur, ChristopherMarto, JoanaBedo, TiborMatsuo, YasumitsuBerteau, Jean-PhilippeMiculescu, MarianBetsch, MarcelMiluski, PiotrBeuran, MirceaMiri, Amir K.Biasetto, LisaMonchau, FrancineBoase, NathanMorris, GordonBodaghi, MahdiMucsi, ZoltánBojić, MirzaMust, IndrekBolamperti, SimonaNa, Hee SamBuxadera-Palomero, JuditNagaoka, ShojiByun, Soo-HwanOtero, AnaCamargo, Samira Esteves AfonsoPadamati, Ramesh BabuCaputi, SergioPaknahad, AliCatapano, GerardoPalazzo, BarbaraCeliz, Adam DavidPan, Guan-TingChen, ChuanruiPatrol, DeliaChen, HongyuPawlik, JustynaChenchev, IvanPellegrini, MarikaCheng, NaichenPeña, Jose IgnacioChereddy, KiranPeng, ShupingChirila, TraianPérez-Álvarez, LeyreChowdhury, Abu Faem Mohammad AlmasPéter, BeatrixChrcanovic, BrunoPiras, CarmenChrzanowski, ŁukaszPopov, AntonCowie, RaelenePrabhakaran, VenkateshkumarCrescenzi, ElviraPreti, GiulioCzeppe, TomaszProchon, MiroslawaDehelean, CristinaQiao, MingyuDeng, YiRadacsi, NorbertDiliën, HanneRaucci, Maria GraziaDinescu, SorinaRichards, Dylan JackDing, JianxunRosati, AlessandraDing, ZhaoyangRoy, Marie E.Dolez, PatriciaRusso, ValentinaDorozhkin, Sergey V.Ryu, Jae-JunDuarte Campos, Daniela FilipaSadowska, BeataEsposito, GennaroSaiz-Poseu, JavierFabregue, DamienSalameh, ChrystelleFang, GangSalayová, AnetaFeng, Sheng-WeiSalerno, MarcoFernández, Juan PedroSammartino, GilbertoFiallo, MarinaSantos-Coquillat, AnaFigueroa López, Kelly JohanaSaratale, Ganesh DattatrayaFilip, AdrianaSaturnino, CarmelaFilipczak, NinaSaville, Stephen P.Fraleoni-Morgera, AlessandroSawicki, JacekFrank-Kamenetskaya, Olga V.Schmidt, FranziskaFrolova, Tatiana S.Scott, Daniel M.Furko, MonikaSfondrini, Maria FrancescaGaidau, CarmenShakouri, AbolfazlGajjar, ChiragSharma, AnuragGashti, Mazeyar ParvinzadehShcharbin, DzmitryGasparski, AlexanderSikder, PrabahaGierszewska, MagdalenaSmolka, MartinGlavina, DomagojSorrentino, AlessandroGlazar, IrenaStan, MirunaGolgovici, FlorentinaStawski, DawidGonçalves, CarlosStingu, Catalina-SuzanaGorseta, KristinaSuárez, MartaGrassia, VincenzoSultana Rekha, RokeyaGrigoraš, Anca GiorgianaTanase, CorneliuGuo, BinTang, Cheng-MingGwiazdowska, DanielaTarasiuk, JolantaHernández, José Carlos RodríguezTecco, SimonaHsiang, Hsing-ITinoco, Arthur D.Iconaru, Simona LilianaTrajkovski, BrankoIordache, FlorinTranquillo, ElisabettaIsola, GaetanoTsai, Ang-ChenIvanov, IvanUlasov, IlyaJain, GauravVelliou, EiriniJaradat, NidalViennot, StéphaneJurak, MałgorzataWajda, AleksandraKaku, MasaruWang, ChaoKarim, LamyaWang, JunshengKarpukhina, NataliaWang, LiqiangKean, ThomasWang, Shi-BinKhurshid, ZohaibWeisel, John W.Kim, JuhyungWiraja, ChristianKim, Young JoXiao, XinxinKlimek, LeszekXiao, ZhigangKoller, MartinYap, Kiryu K.Kolmas, JoannaYazdimamaghani, MostafaKucharska, KarolinaYe, ZhouKuś, WacławYlä-Outinen, LauraKus-Liśkiewicz, MałgorzataYu, JiayiLee, Eun AhYuan, Wei-EnLee, Woo-KyungYue, MinLi, PingZelikman, EvgeniLi, RongpengZhang, WujieLin, Wei-ChunZhou, DongfangLin, WeifengZhou, QihuiLiu, MingxianZille, Andrea

